# Supplementary material for: A paucigranulocytic asthma host environment promotes the emergence of virulent influenza viral variants
Source: eLife. 2021 Feb 16;10:e61803. doi: 10.7554/eLife.61803 (PMC7886327; doi:10.7554/eLife.61803)
Supplement: Supplementary file 1. [file elife-61803-supp1.docx]

**Supplementary Table 1: Forward and reverse primers used for qPCR**

| Gene | Sequence |
| --- | --- |
| *GAPDH* | FW: AGGTGGGTGTGAACGGATTTG RV: TGTAGACCATGTAGTTGAGGTCA |
| *TNF-α* | FW: CATCTTCTCAAAATTCGAGTGACAA RV: TGGGAGTAGACAAGGTACAACCC |
| *OASL2* | FW: GGATGCCTGGGAGAGAATCG RV: TCGCCTGCTCTTCGAAACTG |
| *IL-6* | FW: ACCGCTATGAAGTTCCTCTC RV: CCTCTGTGAAGTCTCCTCTC |
| *IFN-γ* | FW: ACTGGCAAAAGGATGGTGAC RV: TGAGCTCATTGAATGCTTGG |
| *IFN-α* | FW: ACAACAGATCCAGAAGGCTCAAG RV: AGTCTTCCTGGGTCAGAGGAGGTT |
| *MX1* | FW: TCTGAGGAGAGCCAGACGAT RV: ACTCTGGTCCCCAATGACAG |
| Influenza A Matrix | FW: AAGACCAATCCTGTCACCTCTGA RV: TCCTCGCTCACTGGGCA |
