# Supplementary material for: A paucigranulocytic asthma host environment promotes the emergence of virulent influenza viral variants
Source: eLife. 2021 Feb 16;10:e61803. doi: 10.7554/eLife.61803 (PMC7886327; doi:10.7554/eLife.61803)
Supplement: Supplementary file 2. [file elife-61803-supp2.docx]

| Non-asthmatic | | | | Asthmatic | | | |
| --- | --- | --- | --- | --- | --- | --- | --- |
| Day 4 | | **Day 6** | | **Day 4** | | **Day 6** | |
|  |  |  |  |  |  | A139del | 1 |
|  |  |  |  |  |  | A140del | 1 |
|  |  |  |  |  |  | A142del | 1 |
|  |  | A157del | 1 |  |  |  |  |
|  |  | A63del | 1 |  |  |  |  |
|  |  |  |  |  |  | A221R | 1 |
|  |  |  |  |  |  | A240del | 1 |
|  |  |  |  |  |  | A242del | 1 |
|  |  |  |  |  |  | A63del | 1 |
|  |  |  |  |  |  | A643D | 1 |
|  |  |  |  |  |  | A652P | 2 |
|  |  |  |  |  |  | A661E | 1 |
| A741T | 1 |  |  |  |  |  |  |
|  |  |  |  |  |  | A83G | 1 |
|  |  |  |  |  |  | A83P | 1 |
|  |  |  |  |  |  | A83S | 1 |
|  |  | A93P | 1 |  |  |  |  |
|  |  |  |  |  |  | A93T | 1 |
|  |  |  |  |  |  | C610Y | 1 |
|  |  |  |  | C625del | 1 | C625del | 1 |
|  |  |  |  |  |  | C625R | 1 |
|  |  |  |  |  |  | C625S | 2 |
|  |  |  |  | C692I | 1 |  |  |
|  |  |  |  |  |  | C692del | 1 |
|  |  |  |  |  |  | C692G | 1 |
|  |  |  |  |  |  | C692R | 1 |
|  |  |  |  |  |  | C692S | 2 |
|  |  |  |  |  |  | C692T | 1 |
|  |  |  |  |  |  | C693A | 1 |
|  |  |  |  | C693N | 1 | C693N | 2 |
|  |  |  |  | C693R | 1 |  |  |
|  |  |  |  |  |  | C693S | 1 |
|  |  |  |  |  |  | C693Y | 1 |
|  |  |  |  |  |  | C87G | 1 |
|  |  | D120M | 1 |  |  |  |  |
|  |  | D130A | 1 |  |  |  |  |
|  |  |  |  |  |  | D130N | 1 |
|  |  |  |  |  |  | D130V | 1 |
|  |  | D165del | 1 |  |  |  |  |
|  |  | D165N | 1 |  |  |  |  |
|  |  |  |  | D169E | 1 |  |  |
|  |  |  |  |  |  | D230R | 1 |
|  |  |  |  |  |  | D2H | 2 |
|  |  |  |  |  |  | D41stop | 1 |
|  |  |  |  |  |  | D617G | 1 |
|  |  |  |  |  |  | D617Y | 1 |
|  |  |  |  |  |  | D618E | 1 |
|  |  |  |  |  |  | D618stop | 1 |
|  |  |  |  | D619G | 1 |  |  |
|  |  |  |  |  |  | D685A | 1 |
|  |  |  |  |  |  | D685del | 1 |
|  |  |  |  |  |  | D685H | 2 |
|  |  |  |  |  |  | D685Q | 1 |
|  |  |  |  |  |  | D685S | 1 |
|  |  |  |  | D685E | 2 |  |  |
| D729E | 1 |  |  |  |  |  |  |
|  |  |  |  |  |  | D76A | 2 |
|  |  |  |  |  |  | D76H | 2 |
|  |  | E112R | 1 |  |  |  |  |
|  |  | E159del | 1 |  |  |  |  |
|  |  |  |  |  |  | E172P | 1 |
|  |  |  |  | E172Q | 1 |  |  |
|  |  |  |  |  |  | E232C | 1 |
|  |  | E457K | 1 |  |  |  |  |
|  |  |  |  |  |  | E608S | 1 |
|  |  |  |  |  |  | E60A | 1 |
|  |  |  |  |  |  | E60W | 1 |
|  |  |  |  |  |  | E656D | 1 |
|  |  |  |  |  |  | E656Q | 1 |
|  |  |  |  |  |  | E684D | 1 |
|  |  |  |  |  |  | E684del | 1 |
|  |  |  |  |  |  | E684H | 1 |
|  |  |  |  | E686A | 1 |  |  |
|  |  |  |  | E686P | 1 |  |  |
|  |  |  |  |  |  | E684V | 1 |
|  |  |  |  |  |  | E686K | 1 |
|  |  |  |  |  |  | E686Q | 1 |
|  |  |  |  |  |  | E686V | 1 |
| E686T | 1 |  |  |  |  |  |  |
|  |  |  |  |  |  | E697R | 1 |
|  |  |  |  |  |  | E75D | 4 |
|  |  |  |  |  |  | E75V | 2 |
|  |  |  |  |  |  | E78G | 2 |
|  |  |  |  |  |  | E78K | 1 |
|  |  |  |  |  |  | E78Q | 1 |
|  |  |  |  |  |  | E90A | 1 |
|  |  |  |  |  |  | E90del | 1 |
|  |  |  |  |  |  | E90H | 1 |
|  |  |  |  |  |  | E90R | 1 |
|  |  |  |  |  |  | E96D | 1 |
|  |  |  |  |  |  | F150L | 1 |
|  |  |  |  |  |  | F150P | 1 |
|  |  | F166del | 1 |  |  |  |  |
|  |  |  |  |  |  | F696S | 1 |
|  |  |  |  |  |  | F699Y | 1 |
|  |  |  |  |  |  | F700S | 1 |
|  |  |  |  |  |  | G154R | 1 |
|  |  | G125C | 1 |  |  |  |  |
|  |  | G161del | 1 |  |  |  |  |
|  |  |  |  |  |  | G245del | 1 |
|  |  |  |  |  |  | G250del | 1 |
|  |  |  |  |  |  | G37P | 1 |
|  |  |  |  |  |  | G650A | 1 |
| G71R | 1 |  |  |  |  |  |  |
|  |  |  |  |  |  | H184Q | 1 |
|  |  |  |  |  |  | H47T | 1 |
|  |  | I12del | 1 |  |  |  |  |
|  |  | I164del | 1 |  |  |  |  |
|  |  | I205M | 1 |  |  | I205M | 2 |
|  |  |  |  |  |  | I219F | 1 |
|  |  |  |  |  |  | I241del | 1 |
|  |  |  |  |  |  | I248del | 1 |
|  |  |  |  |  |  | I248V | 2 |
|  |  |  |  |  |  | I325V | 1 |
|  |  |  |  |  |  | I606N | 1 |
|  |  |  |  |  |  | I667S | 1 |
|  |  |  |  | I674E | 1 |  |  |
|  |  | I674M | 1 |  |  |  |  |
|  |  |  |  |  |  | I682R | 1 |
|  |  |  |  | I682F | 2 |  |  |
|  |  | I735R | 1 |  |  |  |  |
|  |  |  |  |  |  | I743V | 1 |
|  |  |  |  |  |  | ins212F | 1 |
|  |  |  |  |  |  | ins212M | 1 |
|  |  |  |  |  |  | ins212S | 1 |
|  |  |  |  |  |  | ins214A | 1 |
|  |  |  |  |  |  | ins214G | 1 |
|  |  |  |  |  |  | ins214L | 1 |
|  |  |  |  |  |  | ins214M | 1 |
|  |  |  |  |  |  | ins214R | 1 |
|  |  |  |  |  |  | ins219E | 1 |
|  |  |  |  |  |  | ins219S | 1 |
|  |  | ins458D | 1 |  |  |  |  |
|  |  | ins458L | 1 |  |  |  |  |
|  |  | ins458Q | 1 |  |  |  |  |
|  |  | ins458stop | 1 |  |  |  |  |
|  |  | K11del | 1 |  |  |  |  |
|  |  | K121P | 1 |  |  |  |  |
|  |  | K168del | 1 |  |  |  |  |
|  |  |  |  | K168M | 1 |  |  |
|  |  |  |  |  |  | K188N | 1 |
|  |  |  |  |  |  | K229E | 1 |
|  |  |  |  |  |  | K235Q | 1 |
|  |  | K237R | 1 |  |  |  |  |
|  |  |  |  |  |  | K237H | 1 |
|  |  |  |  |  |  | K52S | 1 |
|  |  | K486L | 1 |  |  |  |  |
|  |  |  |  |  |  | K612V | 1 |
|  |  |  |  |  |  | K653D | 1 |
|  |  |  |  |  |  | K653R | 1 |
|  |  |  |  |  |  | K669N | 1 |
|  |  | K669H | 1 |  |  |  |  |
|  |  |  |  |  |  | K691D | 1 |
|  |  |  |  |  |  | K691G | 1 |
|  |  |  |  | K691S | 1 |  |  |
|  |  |  |  |  |  | K698N | 1 |
|  |  |  |  |  |  | K745R | 1 |
|  |  | L10I | 1 |  |  |  |  |
|  |  | L122del | 1 |  |  |  |  |
|  |  | L122P | 1 |  |  |  |  |
|  |  |  |  |  |  | L133del | 1 |
|  |  |  |  |  |  | L143del | 1 |
|  |  |  |  |  |  | L163P | 1 |
|  |  | L163del | 1 |  |  |  |  |
|  |  | L167del | 1 |  |  |  |  |
|  |  |  |  |  |  | L163Q | 1 |
|  |  |  |  |  |  | L212I | 1 |
|  |  |  |  |  |  | L218M | 1 |
|  |  |  |  |  |  | L222K | 1 |
|  |  |  |  |  |  | L222W | 1 |
|  |  |  |  |  |  | L224T | 1 |
|  |  |  |  |  |  | L224W | 1 |
|  |  |  |  |  |  | L236H | 1 |
|  |  |  |  | L604I | 1 |  |  |
|  |  |  |  |  |  | L611R | 1 |
|  |  |  |  |  |  | L611S | 1 |
|  |  |  |  |  |  | L615del | 1 |
|  |  |  |  | L624del | 1 | L624del | 1 |
|  |  |  |  |  |  | L624S | 1 |
|  |  | L675H | 1 |  |  |  |  |
|  |  |  |  |  |  | L683H | 1 |
|  |  |  |  |  |  | L683P | 1 |
|  |  |  |  | L695I | 2 | L695I | 1 |
|  |  |  |  |  |  | L695V | 1 |
|  |  |  |  |  |  | L695Y | 1 |
|  |  |  |  |  |  | L73Q | 1 |
|  |  |  |  |  |  | L89del | 1 |
|  |  |  |  |  |  | L89M | 1 |
|  |  |  |  |  |  | L89Q | 1 |
|  |  | M111K | 1 |  |  |  |  |
|  |  |  |  | M171I | 1 |  |  |
|  |  |  |  | M174T | 1 |  |  |
|  |  |  |  |  |  | M171R | 1 |
|  |  |  |  |  |  | M171V | 1 |
|  |  |  |  |  |  | M174G | 1 |
|  |  |  |  |  |  | M227E | 1 |
|  |  |  |  |  |  | M227N | 1 |
|  |  |  |  |  |  | M246del | 1 |
|  |  |  |  |  |  | M40Q | 1 |
|  |  |  |  |  |  | M616I | 2 |
|  |  |  |  |  |  | M616S | 1 |
|  |  |  |  |  |  | M655I | 1 |
|  |  |  |  |  |  | M655R | 1 |
|  |  |  |  |  |  | M688del | 1 |
|  |  |  |  | M688I | 1 | M688I | 2 |
|  |  |  |  | M688T | 1 |  |  |
|  |  |  |  | M688V | 1 |  |  |
| M688W | 1 |  |  |  |  |  |  |
|  |  |  |  |  |  | M744I | 1 |
|  |  | N105D | 1 |  |  |  |  |
|  |  |  |  |  |  | N134del | 1 |
|  |  |  |  |  |  | N136del | 1 |
|  |  | N158del | 1 |  |  |  |  |
|  |  |  |  |  |  | N158K | 1 |
|  |  |  |  | N175D | 1 |  |  |
|  |  |  |  |  |  | N225T | 1 |
|  |  |  |  |  |  | N328L | 1 |
|  |  |  |  |  |  | N44H | 1 |
|  |  | N455H | 1 |  |  |  |  |
|  |  | N485S | 1 |  |  |  |  |
|  |  |  |  |  |  | N518K | 1 |
|  |  |  |  |  |  | N58D | 1 |
|  |  |  |  |  |  | N58H | 1 |
|  |  |  |  |  |  | N58R | 1 |
|  |  |  |  |  |  | N58S | 1 |
|  |  | N58del | 1 |  |  |  |  |
|  |  |  |  | N603A | 1 |  |  |
| N603T | 1 |  |  |  |  |  |  |
|  |  |  |  |  |  | N641T | 1 |
| N641D | 1 |  |  |  |  |  |  |
|  |  |  |  |  |  | N642H | 1 |
|  |  | N676T | 1 |  |  |  |  |
|  |  |  |  |  |  | N671K | 1 |
|  |  |  |  |  |  | N694K | 1 |
|  |  |  |  |  |  | N694Q | 1 |
|  |  |  |  | N694E | 1 |  |  |
|  |  |  |  | N694R | 1 | N694R | 2 |
|  |  |  |  |  |  | N77I | 2 |
|  |  |  |  |  |  | N77T | 1 |
|  |  |  |  |  |  | N77V | 2 |
|  |  | P13del | 1 |  |  |  |  |
|  |  |  |  |  |  | P138del | 1 |
|  |  |  |  |  |  | P244del | 1 |
|  |  |  |  |  |  | P607G | 1 |
| P627H | 1 |  |  |  |  |  |  |
|  |  | P630T | 1 |  |  |  |  |
|  |  | P64I | 1 |  |  |  |  |
|  |  |  |  |  |  | P651Q | 1 |
|  |  | P668H | 1 |  |  |  |  |
|  |  |  |  |  |  | P668Q | 1 |
|  |  |  |  |  |  | P668T | 1 |
|  |  |  |  |  |  | P701H | 1 |
|  |  |  |  |  |  | P72A | 1 |
|  |  |  |  |  |  | P72S | 1 |
|  |  |  |  |  |  | P74A | 2 |
|  |  |  |  |  |  | P79H | 1 |
|  |  |  |  |  |  | P79L | 1 |
|  |  | Q115L | 1 |  |  |  |  |
|  |  |  |  |  |  | Q115I | 1 |
|  |  |  |  |  |  | Q115T | 1 |
|  |  | Q116stop | 1 |  |  |  |  |
|  |  | Q124H | 1 |  |  |  |  |
|  |  | Q124R | 1 |  |  |  |  |
|  |  | Q127stop | 1 |  |  |  |  |
|  |  |  |  |  |  | Q127L | 1 |
|  |  |  |  |  |  | Q127N | 1 |
|  |  |  |  |  |  | Q137del | 1 |
|  |  |  |  |  |  | Q137K | 1 |
|  |  | Q186H | 1 |  |  |  |  |
|  |  |  |  |  |  | Q210A | 1 |
|  |  |  |  |  |  | Q247del | 1 |
|  |  | Q460K | 1 |  |  |  |  |
|  |  | Q65stop | 1 |  |  |  |  |
|  |  |  |  | Q687A | 1 | Q687A | 1 |
|  |  |  |  | Q687E | 1 |  |  |
| Q687P | 1 |  |  |  |  |  |  |
|  |  |  |  |  |  | Q687L | 1 |
|  |  |  |  |  |  | Q690P | 1 |
|  |  |  |  | Q690S | 2 |  |  |
|  |  |  |  |  |  | Q84stop | 1 |
|  |  | R118M | 1 |  |  |  |  |
|  |  |  |  |  |  | R118L | 1 |
|  |  |  |  |  |  | R135del | 1 |
|  |  |  |  |  |  | R151S | 1 |
|  |  | R162del | 1 |  |  |  |  |
|  |  |  |  |  |  | R162K | 1 |
|  |  |  |  |  |  | R211P | 1 |
|  |  |  |  |  |  | R215K | 1 |
|  |  |  |  |  |  | R220E | 1 |
|  |  |  |  |  |  | R220K | 1 |
|  |  |  |  |  |  | R233G | 1 |
|  |  | R238G | 1 |  |  |  |  |
|  |  |  |  |  |  | R239del | 1 |
|  |  |  |  |  |  | R249del | 1 |
|  |  |  |  |  |  | R327K | 1 |
|  |  |  |  |  |  | R45T | 1 |
|  |  | R571M | 1 |  |  |  |  |
|  |  |  |  |  |  | R621P | 2 |
|  |  |  |  |  |  | R621Q | 1 |
|  |  |  |  |  |  | R621S | 1 |
|  |  |  |  |  |  | R623K | 1 |
|  |  |  |  |  |  | R623S | 1 |
|  |  |  |  | R623del | 1 |  |  |
|  |  |  |  | R623W | 1 |  |  |
|  |  | R670G | 1 |  |  |  |  |
|  |  | R672S | 1 |  |  |  |  |
|  |  |  |  |  |  | R672W | 1 |
|  |  |  |  | R672G | 1 |  |  |
| R672P | 2 |  |  |  |  |  |  |
| R727T | 1 |  |  |  |  |  |  |
|  |  | R734S | 1 |  |  |  |  |
|  |  | S160del | 1 |  |  |  |  |
|  |  |  |  |  |  | S160A | 1 |
|  |  |  |  |  |  | S173K | 1 |
|  |  |  |  |  |  | S173stop | 1 |
|  |  |  |  |  |  | S173T | 1 |
|  |  |  |  | S173Y | 1 |  |  |
|  |  |  |  |  |  | S50R | 1 |
|  |  |  |  |  |  | S654I | 1 |
|  |  |  |  |  |  | S654N | 2 |
|  |  |  |  |  |  | S654Y | 1 |
|  |  |  |  |  |  | S665T | 1 |
| S673P | 1 |  |  |  |  |  |  |
|  |  | S741T | 4 |  |  |  |  |
|  |  |  |  |  |  | S702N | 1 |
|  |  |  |  |  |  | S703R | 1 |
|  |  |  |  |  |  | S704T | 1 |
|  |  |  |  | S712Y | 1 |  |  |
|  |  |  |  | S713R | 1 |  |  |
|  |  |  |  |  |  | S80H | 2 |
|  |  |  |  |  |  | S80K | 1 |
|  |  |  |  |  |  | S80R | 1 |
|  |  |  |  |  |  | T117S | 1 |
|  |  | T123A | 1 |  |  |  |  |
|  |  | T128K | 1 |  |  |  |  |
|  |  |  |  |  |  | T128H | 1 |
|  |  | T132stop | 1 |  |  |  |  |
|  |  |  |  |  |  | T141del | 1 |
|  |  |  |  |  |  | T223R | 1 |
|  |  |  |  |  |  | T228S | 1 |
|  |  |  |  |  |  | T243del | 1 |
|  |  |  |  |  |  | T326C | 1 |
|  |  | T46I | 1 |  |  |  |  |
|  |  |  |  |  |  | T46N | 1 |
|  |  | T487P | 1 |  |  |  |  |
|  |  |  |  |  |  | T56V | 1 |
|  |  | T56R | 1 |  |  |  |  |
|  |  |  |  |  |  | T57D | 1 |
|  |  |  |  |  |  | T57Q | 1 |
|  |  |  |  |  |  | T57R | 1 |
|  |  |  |  |  |  | T57S | 1 |
|  |  | T570P | 1 |  |  |  |  |
|  |  | T57M | 1 |  |  |  |  |
|  |  | T59del | 1 |  |  |  |  |
|  |  |  |  |  |  | T59A | 1 |
|  |  |  |  |  |  | T59L | 1 |
|  |  |  |  |  |  | T59S | 1 |
|  |  | T61A | 1 |  |  |  |  |
|  |  |  |  |  |  | T61I | 1 |
|  |  | T662P | 1 |  |  |  |  |
|  |  |  |  |  |  | T663E | 1 |
|  |  |  |  | T677A | 1 |  |  |
|  |  | V113L | 1 |  |  |  |  |
|  |  | V114I | 1 |  |  |  |  |
|  |  |  |  |  |  | V43E | 1 |
|  |  |  |  |  |  | V609del | 1 |
|  |  |  |  |  |  | V644L | 1 |
|  |  | V645I | 1 |  |  |  |  |
|  |  |  |  |  |  | V645F | 1 |
|  |  |  |  |  |  | V645M | 1 |
|  |  |  |  |  |  | V645R | 1 |
|  |  |  |  |  |  | V88A | 1 |
|  |  |  |  |  |  | V88D | 1 |
|  |  |  |  |  |  | V88del | 1 |
|  |  |  |  |  |  | W131stop | 1 |
|  |  | W55V | 1 |  |  |  |  |
|  |  |  |  |  |  | W55del | 1 |
|  |  |  |  |  |  | W55Q | 1 |
|  |  |  |  |  |  | W613R | 1 |
|  |  |  |  |  |  | W666N | 1 |
|  |  |  |  |  |  | W666S | 1 |
|  |  |  |  |  |  | Y129F | 1 |
|  |  | Y129H | 1 |  |  |  |  |
|  |  | Y129N | 1 |  |  |  |  |
|  |  |  |  |  |  | Y129L | 1 |
|  |  |  |  |  |  | Y324E | 1 |
|  |  |  |  |  |  | Y38W | 1 |
|  |  | Y49D | 1 |  |  |  |  |
|  |  |  |  |  |  | Y49K | 1 |
|  |  |  |  |  |  | Y557H | 1 |
|  |  |  |  |  |  | Y620K | 1 |
|  |  |  |  |  |  | Y620L | 1 |
|  |  |  |  |  |  | Y657H | 2 |
|  |  |  |  |  |  | Y657N | 1 |
|  |  |  |  |  |  | Y657P | 1 |
|  |  |  |  |  |  | Y689E | 1 |
|  |  |  |  |  |  | Y689H | 1 |
|  |  |  |  |  |  | Y689N | 1 |
|  |  |  |  |  |  | Y689R | 2 |
|  |  |  |  | Y689C | 1 |  |  |
|  |  |  |  | Y689L | 1 |  |  |
|  |  |  |  |  |  | Y705D | 1 |
|  |  |  |  |  |  | Y82F | 1 |
|  |  |  |  |  |  | Y82N | 1 |
