# Supplementary figures and images for: A paucigranulocytic asthma host environment promotes the emergence of virulent influenza viral variants
Source: eLife. 2021 Feb 16;10:e61803. doi: 10.7554/eLife.61803 (PMC7886327; doi:10.7554/eLife.61803)

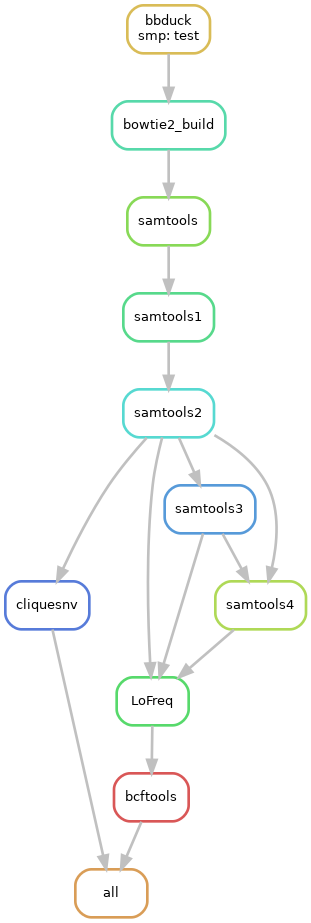

Supplement: Supplementary file 3. [file elife-61803-supp3.png]
